# Quantitative Multicolor Compositional Imaging Resolves Molecular Domains in Cell-Matrix Adhesions

**DOI:** 10.1371/journal.pone.0001901

**Published:** 2008-04-02

**Authors:** Eli Zamir, Benjamin Geiger, Zvi Kam

**Affiliations:** Department of Molecular Cell Biology, The Weizmann Institute of Science, Rehovot, Israel; University of Birmingham, United Kingdom

## Abstract

**Background:**

Cellular processes occur within dynamic and multi-molecular compartments whose characterization requires analysis at high spatio-temporal resolution. Notable examples for such complexes are cell-matrix adhesion sites, consisting of numerous cytoskeletal and signaling proteins. These adhesions are highly variable in their morphology, dynamics, and apparent function, yet their molecular diversity is poorly defined.

**Methodology/Principal Findings:**

We present here a compositional imaging approach for the analysis and display of multi-component compositions. This methodology is based on microscopy-acquired multicolor data, multi-dimensional clustering of pixels according to their composition similarity and display of the cellular distribution of these composition clusters. We apply this approach for resolving the molecular complexes associated with focal-adhesions, and the time-dependent effects of Rho-kinase inhibition. We show here compositional variations between adhesion sites, as well as ordered variations along the axis of individual focal-adhesions. The multicolor clustering approach also reveals distinct sensitivities of different focal-adhesion-associated complexes to Rho-kinase inhibition.

**Conclusions/Significance:**

Multicolor compositional imaging resolves “molecular signatures” characteristic to focal-adhesions and related structures, as well as sub-domains within these adhesion sites. This analysis enhances the spatial information with additional “contents-resolved” dimensions. We propose that compositional imaging can serve as a powerful tool for studying complex multi-molecular assemblies in cells and for mapping their distribution at sub-micron resolution.

## Introduction

Molecular processes in cells involve multiple components that are dynamically interacting with each other. The characterization of such events requires, therefore, a capability to simultaneously localize and quantify the relevant molecules at the maximal resolution. A wide range of methods were developed for measuring the levels of a large number of molecules, biochemically or optically[1]–[6], yet these approaches lack the sub-cellular spatial information. In this study we developed a light-microscopy-based imaging approach which defines multi-component compositions at single pixel resolutions, and applied novel analysis for defining specific “molecular signatures” and visualizing their sub-cellular distributions.

Compositional imaging was applied here to study the molecular reorganization of cell-matrix adhesions in rat embryo fibroblasts (REF52). The assembly and modulation of cell adhesions are highly regulated dynamic processes, characterized by the selective recruitment of specific subsets of molecules, derived from a repertoire of over 150 proteins, including transmembrane receptors (primarily different integrins), cytoskeletal and adapter proteins (such as actin, vinculin, paxillin, zyxin and α-actinin) and enzymes (such as focal-adhesion kinase, FAK)[7], [8]. A fundamental feature of cell-matrix adhesions is the high diversity in their molecular composition and dynamics, which was studied by simultaneous two-component labeling of fixed cells, time-resolved experiments with GFP-tagged adhesion components and time-lapse movies of cells fixed and labeled at the end-point[7]–[12]. Based on morphological and molecular criteria, several types of cell-matrix adhesions were distinguished in cultured cells. These include focal-complexes, which are small and short-lived contacts formed at the edge of the lamellipodium, focal-adhesions, which are associated with the ends of contractile actin stress-fibers, elongated fibrillar-adhesions, which play a role in extracellular matrix fibrillogenesis, and “3D matrix adhesions”, which are formed with pre-assembled matrix fibrils. Each of these forms is characterized by distinct dynamic properties, stability, mechanosensitivity and molecular composition[7], [9]–[11]. However, the relationships between these functional and molecular features are still poorly understood. A major reason is the presence of composition heterogeneity between adhesions of apparently the same type, and within a single adhesion structure, and the lack of tools for exploring and visualizing these multi-component compositions at high spatial resolutions in relation to cellular behavior. Here we show that compositional imaging provides such a powerful tool for exploring the molecular diversity of focal-adhesions, both in control cells and following modulation of Rho-kinase activity.

Compositional imaging of focal-adhesions was obtained by simultaneous labeling of cells for different combinations of 5 focal-adhesion components, and acquiring the corresponding 5-color images. Multi-dimensional cluster analysis of the pixels of these images identifies typical “compositional signatures” found in the adhesion sites of the labeled cells. Coloring pixels according to these signatures revealed their unique organization between and within spatial sub-domains of focal-adhesions and stress-fibers. We further show that cellular perturbations, such as modulation of actomyosin contractility by Rho-kinase inhibition, differentially affect the abundance and distribution of the various signatures along focal-adhesions and stress-fibers. We discuss here the implications of this compositional mapping for characterizing focal-adhesion assembly, and its general applicability for molecular characterization of complex sub-cellular structures.

## Results

### Resolving compositional signatures

**Figure 1:**
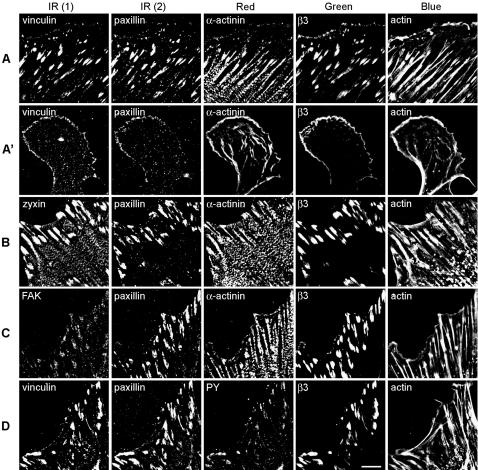
Images of REF52 cells labeled for four sets of five focal-adhesion-associated components. REF52 cells, stably expressing β_3_-integrin-GFP, were fixed 24 hours after plating and labeled for actin and paxillin, as well as for: (A) vinculin and α-actinin, (B) zyxin and α-actinin, (C) FAK and α-actinin, (D) vinculin and phosphotyrosine (PY). (A') REF52 cells treated for 3 hours with 100 µM of the Rho-kinase inhibitor Y-27632 and labeled as in (A). The fluorophores used here include: Cy5 (for the IR1 channel), Alexa-750 (IR2), Cy3 or Alexa-555 (red), GFP (green) and CPITC (blue). Images were acquired using selective excitation and emission filter sets for five fluorescent channels. Scale bar, 10 µm.

REF52 cells, stably expressing β_3_-integrin tagged with EGFP (β_3_-integrin-GFP), were fluorescently labeled for 4 additional focal-adhesion-associated molecules. Altogether, 8 molecules (actin, β_3_-integrin, paxillin, α-actinin, vinculin, FAK, zyxin and phosphotyrosine) were visualized using 4 labeling sets (A–D, as defined in Fig. 1 and Table 1). This labeling was applied to well-spread, untreated fibroblasts, to cells treated with the Rho-kinase inhibitor Y-27632 and to cells at 3, 15 or 60 minutes after washout of the drug and recovery from this treatment. The effect of this inhibitor is shown in Figure 1, for one set of labeled components.

**Table 1:** Multicolor labeling scheme.

color	set	staining		target
Blue	A	CPITC-phalloidin^a^		actin
	B			
	C			
	D			
Green	A	β_3_-integrin-GFP (stable transfection)		β3-integrin
	B			
	C			
	D			
Red	A	Mouse IgM anti-α-actinin^b^, followed by an isotype-specific Cy3-conjugated goat anti mouse-IgM^c^		α-actinin
	B			
	C			
	D	Mouse IgG anti-PY^d^ complexed with Alexa-555-Fab^e^		PY
IR1	A	Mouse IgG anti-paxillin^f^ complexed with Alexa-750-conjugated Fab fragments^g^		paxillin
	B			
	C			
	D			
IR2	A	Rabbit IgG anti-vinculin^h^	followed by Cy5-conjugated goat-anti-rabbit IgG	vinculin
	B	Rabbit IgG anti-zyxin^i^		zyxin
	C	Rabbit IgG anti-FAK^j^		FAK
	D	Rabbit IgG anti-vinculin^h^		vinculin

a CPITC-conjugated phalloidin (Sigma-Aldrich Co.);

b mouse IgM anti-α-actinin primary antibody (clone 75.2, catalogue number A5044, Sigma-Aldrich Co.);

c isotype-specific Cy3-conjugated goat anti mouse-IgM secondary antibody;

d mouse IgG anti-phosphotyrosine antibody (PT66, Sigma-Aldrich Co.);

e Alexa-555-conjugated Fab fragments (Zenon kit, Molecular Probes Inc.);

f mouse IgG anti-paxillin antibody (Transduction Laboratories, Lexington, KY, USA);

g Alexa-750-conjugated Fab fragments (Zenon kit, Molecular Probes Inc., Eugene, OR, USA);

h rabbit anti-vinculin antibody (clone R695 [34]);

i rabbit-anti-zyxin antibody (B71, kindly provided by Laura Hoffman and Mary Beckerle, Huntsman Cancer Institute and Department of Biology, University of Utah, Salt Lake City, UT, USA);

j rabbit anti-FAK antibody (AHO0502, Biosource International Inc., CA, USA).

**Figure 2:**
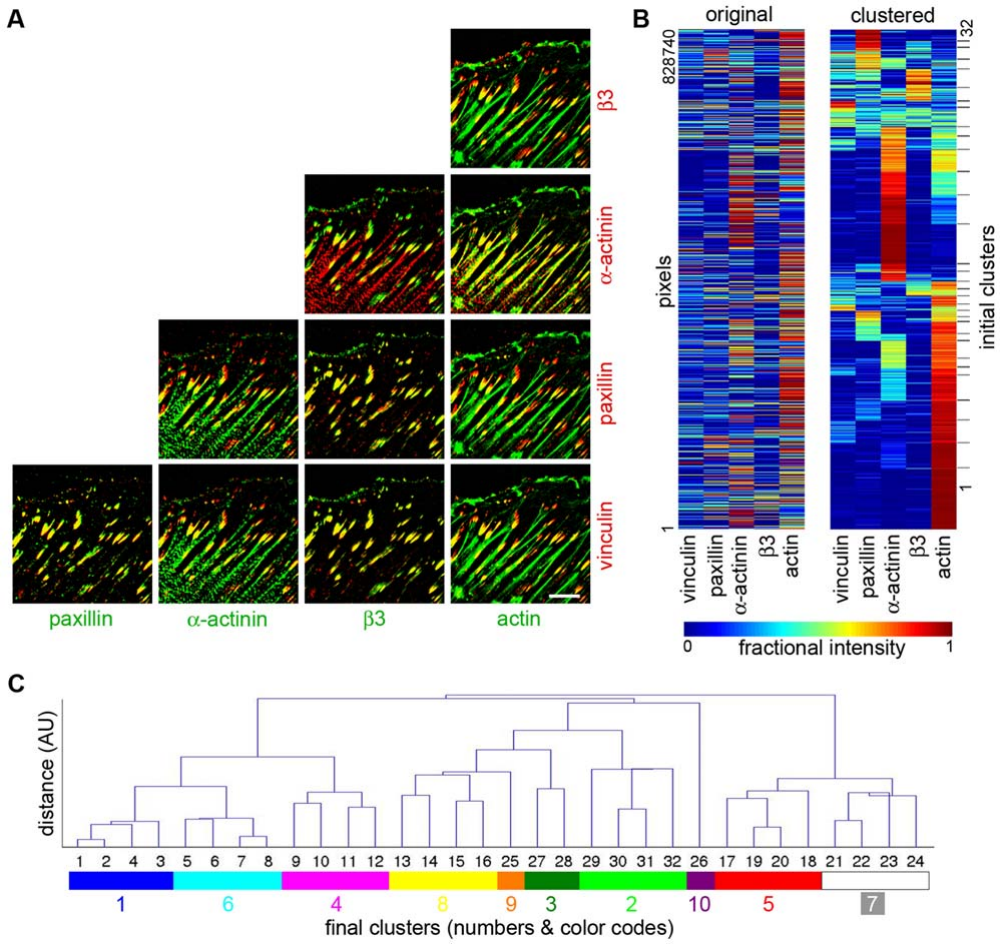
Identifying compositional signatures of adhesion sites by cluster analysis. REF52 cells were subjected to: no treatment, Y-27632, and Y-27632 with a subsequent recovery period of 3, 15 or 60 minutes. The cells were then fixed, labeled for vinculin, paxillin, α-actinin and actin, and their 5-color images (including β_3_-integrin-GFP) were analyzed. For each of the 5 treatments 4 cells were sampled, creating a pool of 20 cells. (A) Images showing a single non-treated, 5-color-labeled, cell in all 10 possible combinations of 2 components superposition images. Scale bar, 10 µm. (B) A matrix presenting the composition of all pixels above background in the 20 multicolor images (original). Color indicates the fractional intensity of a given component in each given pixel. The rows were then reordered according to the top-down clustering algorithm[24] based on compositional similarity (clustered). The process was deliberately designed to over-divide the data into 32 clusters. (C) Bottom-up merging of the over-divided clusters. The dendrogram presents the hierarchical distance between the merged clusters during the merging process. The significance of each cluster along the merging process (i.e. each node in the dendrogram) was further evaluated visually, based on the spatial coherence of the sub-cellular distribution of its pixels. Thus, the initial 32 clusters were merged to 10 final ones, which define compositional signatures, and were assigned distinguishable colors for visualizing their sub-cellular distribution.

**Figure 3:**
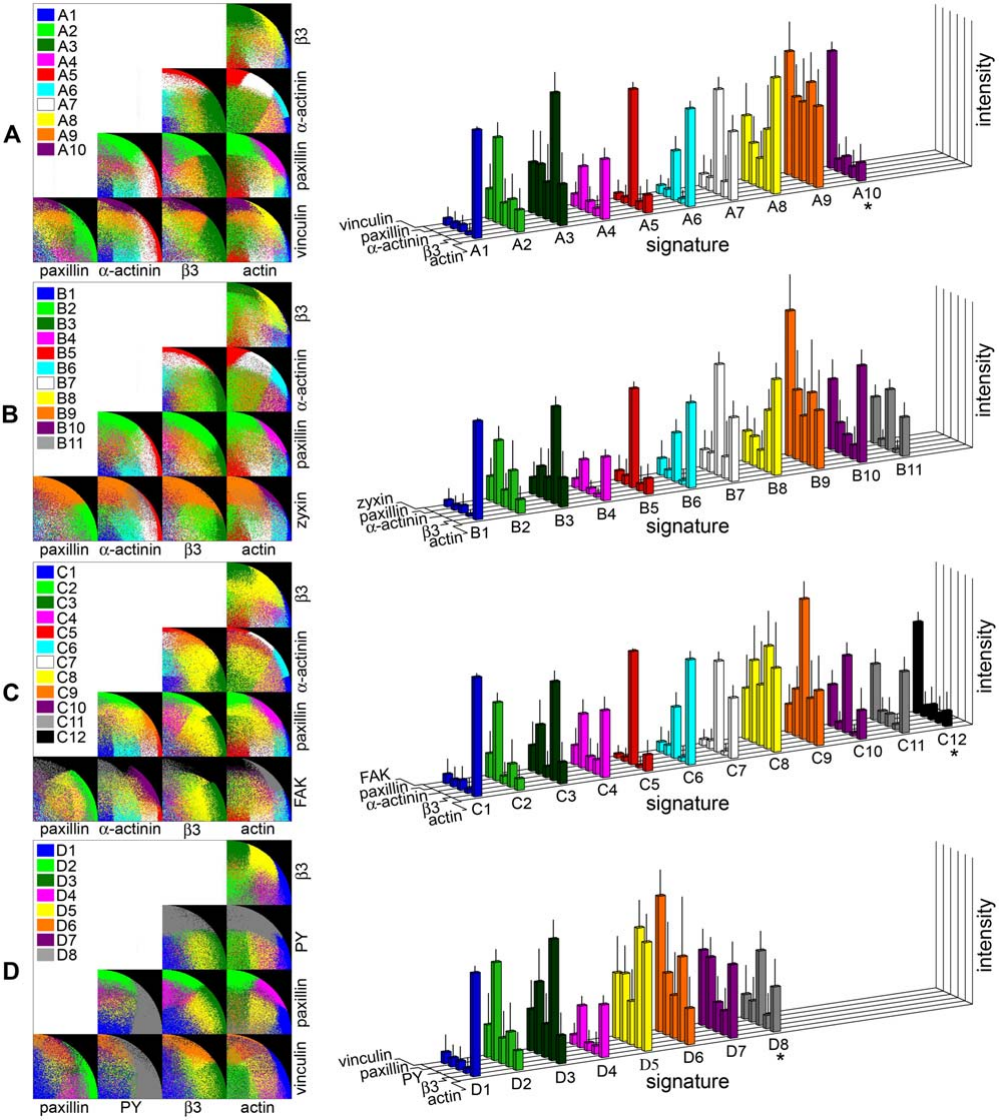
Five-component compositional signatures of cell-matrix adhesions and stress-fibers. The process for defining compositional signatures, as shown in Figure 2, was performed for four labeling sets (A–D) of five components each (see Fig. 1 legend). Right, bar-plots presenting the average intensity for each component in each cluster, defining the compositional signatures. These intensities are scaled, but not normalized to one-unit composition vector (V_s_, see Materials and Methods section), to allow comparison of actual labeling intensities between signatures. Clusters from different labeling sets, which have similar composition for the shared components, were given the same number and color. The asterisk symbol indicates clusters that were later defined as noise based on their spatial distribution in the cells. The standard deviations were calculated as described in the text. Left, scatter-plots presenting, for each labeling set, the fractional intensity of 2 components (out of 5-components composition) in each pixel, for all possible 2-from-5 combinations. Each dot in the scatter-plots corresponds to a pixel, colored according to the cluster it is assigned to. Due to the normalization to one-unit composition vector (see Materials and Methods section), at each two-components projection the pixels cannot span more than a quarter-circle with a radius of one, but can be at smaller radiuses due to the other 3 components excluded from the projection.

Multicolor data indicates the levels of the labeled components (5 for this data set) at a single pixel resolution. It is, however, practically impossible to comprehend the complex relationships between all 5 components by direct visual comparison of the images of the separate components (as apparent from Fig. 1), by superimposing all possible two- or three-color combinations (Fig. 2A), or by superimposing all 5 colors (Supplementary Fig. S7). Quantitative multi-parametric approach is therefore essential to extract and visualize composition information from the multicolor data. Towards that end, we clustered the pixels of the multicolor data based on multicolor composition similarity (Fig. 2). This was performed on a pool containing all pixels, positively labeled for at least one component, from images of 4 cells for each of the 5 treatments (untreated cells, Y-27632-treated cells, and Y-27632-treated cells followed by 3 recovery times, altogether 20 cells for each labeling combination). Each of the resulting clusters (Fig. 2 and Supplementary Fig. S5) defines a distinct compositional signature (Fig. 3 right and Supplementary Table S1). In order to compare fluorescent levels in the different signatures we calculated the averaged color-scaled vectors, V_s_ (see Materials and Methods). For convenience, the clusters and the corresponding compositional signatures were named with a letter identifying the labeling set from which they were resolved (A–D), and a number such that signatures of different labeling sets that are similar (in respect to the shared components of these sets) carry the same number (Fig. 3 and Table 2). For example, signatures A1, B1, C1 and D1 contain actin, with very low levels of the other tested components (Fig. 3, right). It should nevertheless be noted that similar signatures in the four sets should not be considered identical, since they may differ in the level of components that are not shared. Since the standard deviations of the non-normalized vectors reflect the variations in intensity, (not in compositions), error bars in Fig. 3 were calculated from the standard deviation of the normalized vectors and multiplied by the cluster-averaged ratio between the non-normalized and the normalized values. This is an accurate estimate, based on the central limit theorem.

**Table 2:** Localization and abundance of composition signatures in non-treated REF52 cells.

		common feature	labeling set			
			A	B	C	D
**signature**	**1**	roughly only actin	SF	SF	SF	SF
			31%	32%	48%	54%
	**2**	high levels of paxillin	AD	AD	AD	AD
			14%	5%	3%	12%
	**3**	high levels of β3-integrin	AD	---	AD	AD
			3%	0%	2%	5%
	**4**	high levels of actin and paxillin	AD+SF	SF	AD+SF	AD+SF
			9%	5%	4%	11%
	**5**	sets A, B and C: roughly only α-actinin with low levels of actin	SF	SF	SF	AD
			8%	7%	3%	4%
	**6**	sets A, B and C: actin and α-actinin (higher actin-to-α-actinin ratio)	SF	SF	SF	AD
			13%	12%	15%	8%
	**7**	sets A, B and C: actin and α-actinin (lower actin-to-α-actinin ratio)	SF	SF	SF	AD+SF
			8%	4%	3%	6%
	**8**	no common feature	AD	AD	AD	*
			8%	1%	13%	
	**9**	no common feature	AD	AD	---	*
			5%	20%	1%	
	**10**	no common feature	*	AD+SF	---	*
				9%	1%	
	**11**	no common feature	*	SF	SF	*
				5%	6%	

The abundance of each signature is shown as the percentage of pixels with that signature from all the clustered pixels of the non-treated cells in a given labeling set. The localization of the signatures in stress-fibers (SF) and adhesion sites (AD), or their absence from these structures (---), is indicated. Asterisks indicate letter-number combinations that do not correspond to a defined signature (according to Fig. 3), or that correspond to signatures defined as noise (see Fig. 3).

The importance of resolving the clusters based on all 5 components simultaneously can be appreciated by examining all possible two-component projections of the clusters composition (Fig. 3, left). Clearly, the composition clusters resolved using 5 components become highly superimposed, and therefore irresolvable, when projected on two-component combinations. Noteworthy, although five components could have potentially give rise to a huge number of compositions (*N^5^* where *N* is the number of intensity variations resolved for each component), the number of different compositions that actually exist in cells is much smaller, in the range of 10. Thus, this analysis identifies which compositions are present, out of a much larger repertoire of possible compositions.

### Mapping compositional signatures

**Figure 4:**
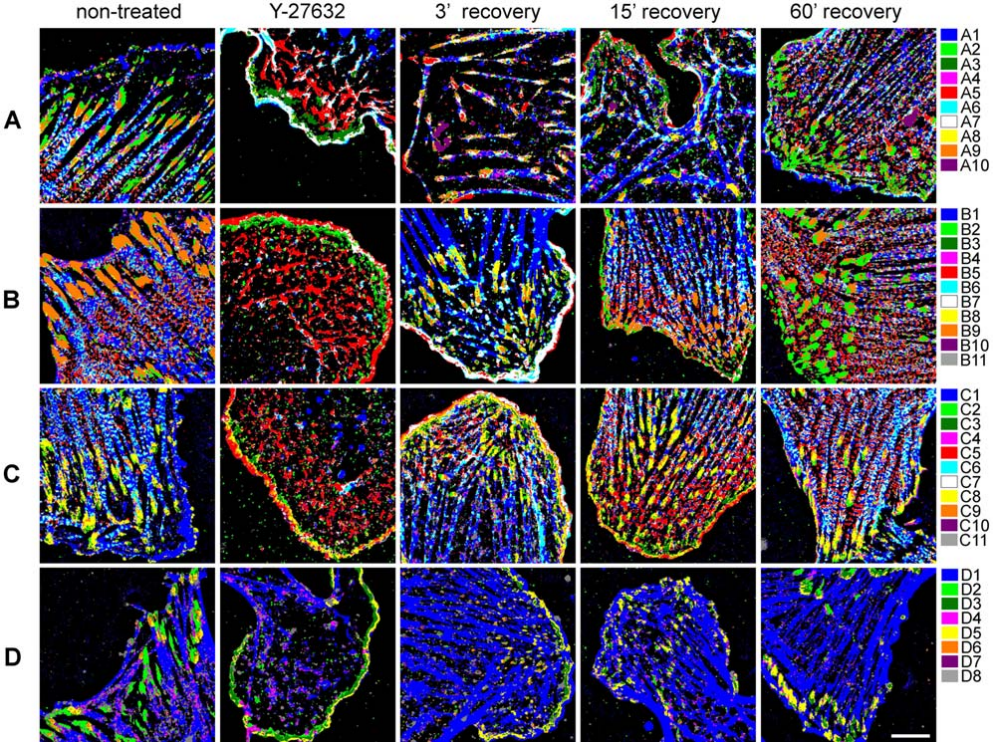
Sub-cellular localization of the compositional clusters in non-treated and treated REF52 cells. Images of the 5-components-labeled cells, in which each pixel is colored according to its cluster assignment (the color-code is indicated on the right, and is consistent with Fig. 3). The rows correspond to the four labeling sets (A–D), as shown in Figure 3. The columns correspond to treatments (non-treated, Y-27632 treatment, and Y-27632 treatment with a subsequent recovery period of 3, 15 and 60 minutes). Scale bar, 10 µm.

Given the signature assignment for each pixel, it is possible to visualize the sub-cellular distribution of the different compositions in REF52 cells by coloring each pixel according to its cluster identity (Fig. 4 and Supplementary Figs. S1, S2, S3, and S4). Big part of these signatures is mapped in non-treated cells exclusively to either adhesion sites or stress-fibers (Table 2). In addition, the compositional analysis reveals sub-domains with distinct compositions located within these structures. Specifically, signatures that are enriched with paxillin and contain low levels of actin (A2, B2, C2 and D2) are located at the edge of focal-adhesions (Fig. 4 and Supplementary Figs. S1, S2, S3 and S4). In labeling set B, the signature containing high level of zyxin together with paxillin, α-actinin, β3 and actin (B9) is found in focal-adhesions, but apparently absent from focal-complexes, which are dominated by signature B2 (low zyxin, see Fig. 4 and Supplementary Fig. S2). This is consistent with previous findings about the localization of zyxin[12]. In labeling sets that contain vinculin (sets A and D) a high heterogeneity exists between cells regarding the abundance of signatures A2, A3, A8 and A9 in focal-adhesions (Supplementary Fig. S6). Yet, the relative spatial relations between signatures localized along the focal-adhesion is preserved, such that A2 is located in the distal edge of the focal-adhesions, followed by A3, then A9 and then A8, which is located at the edge proximal to the stress-fibers. It is noteworthy that, in contrast to the diversity between focal-adhesions of different cells, a remarkable similarity was noted between focal-adhesions located in the same cell, and particularly in the same sub-cellular region (Supplementary Fig. S6). This suggests that both global cellular states and local factors can modulate various types of focal-adhesion assembly, without altering the spatial organization of distinct sub-domains within focal-adhesions.

### Effects of Rho-kinase inhibition on compositional signatures

**Figure 5:**
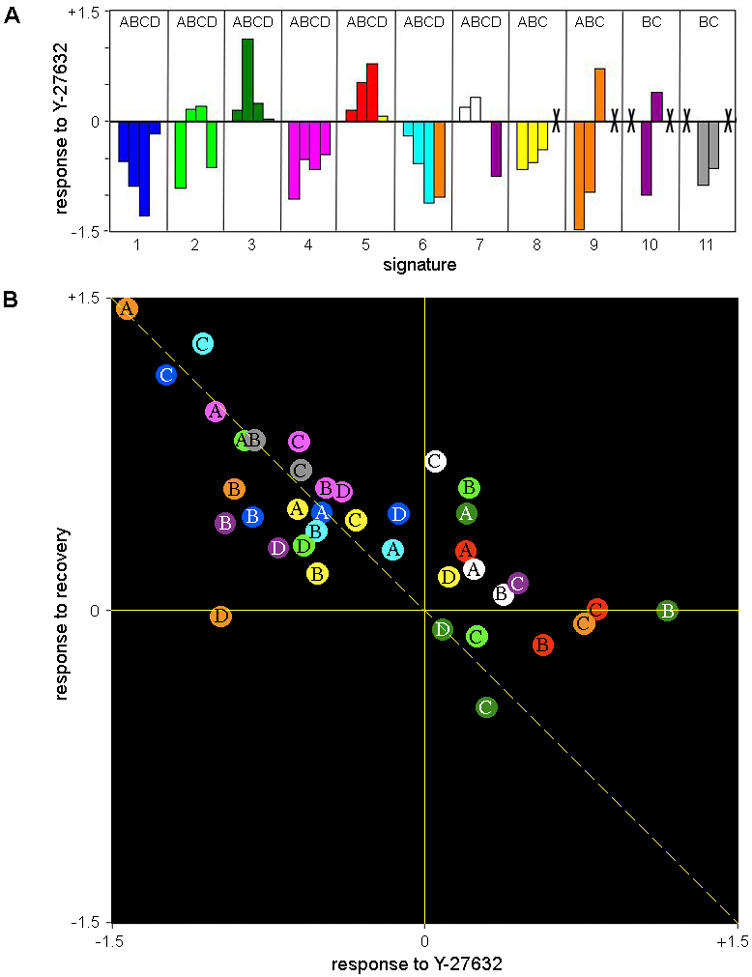
The effect of Rho-kinase inhibition and recovery on the abundance of compositional signatures. (A) The number of pixels assigned to each signature was counted in non-treated cells and in cells treated with Y-27632 (based on Fig. 4 and Supplementary Figs. S1, S2, S3 and S4). The response of each signature to Y-27632 was then defined as: Log(number of pixels in Y-27632 treated cells/number of pixels in non-treated cells). Thus, positive values indicate increase in abundance, negative values indicate decrease and zero indicates no change, in response to Y-27632. Crosses exclude letter-number combinations that do not correspond to a defined signature (according to Fig. 3), or that correspond to signatures defined as noise (see Fig. 3) or signatures absent in non-treated cells (see Table 2). (B) Changes in the abundance of compositional signatures in response to Rho-kinase inhibition and following its recovery. Each position of each signature in the scatter plot is determined by its response to the Y-27632 treatment (horizontal axis) and to the recovery treatment (vertical axis). The response to Y-27632 was calculated as described for (A). The response to recovery was calculated as Log(number of pixels after recovery of 60 minutes/number of pixels in Y-27632 treated cells). The signatures are marked with a letter A–D, indicating the particular labeling set, and a color indicating its number, consistently with Figure 3. The diagonal dashed line marks the expected trend if the response to Y-27632 and the recovery were exactly opposite processes.

Addition of the Rho-kinase inhibitor Y-27632 to well-spread REF52 cells has a profound effect on the actin cytoskeleton and the associated matrix adhesions. The changes observed include the destruction of stress-fibers, loss of focal-adhesions, and development of large lamellipodia enriched with focal-complexes and α-actinin/actin-rich meshwork (Fig. 4, and Supplementary Figs. S1, S2, S3 and S4). Examination of the distribution of the various compositional signatures enabled to dissect and monitor the differential sensitivities of the various molecular complexes to perturbation altering mechanical forces exerted by actin (Figs. 4, 5 and Supplementary Figs. S1, S2, S3 and S4). Indeed, some signatures were very sensitive to Rho-kinase inhibition, while others were resistant or even enhanced by it (Fig. 5) and reorganized into elongated structures (Fig. 4 and Supplementary Figs. S1, S2, S3 and S4).

Compositions dominated by actin, either alone (A1, B1, C1, D1) or with additional protein (e.g. paxillin (A4, B4, C4, D4), FAK (C11), α-actinin (A6, B6, C6) zyxin (B10) or α-actinin and zyxin (B11) or with β_3_-integrin and other components (A8, B8, C8)) were highly sensitive to Rho-kinase inhibition (Fig. 5). Compositions with high levels of zyxin (B9, B10, B11) or vinculin (A9, D6) were also reduced upon Y-27632 treatment. On the other hand, compositions dominated by α-actinin (A5, B5, C5), or α-actinin with some actin (A7, B7, C7), were resistant or even enhanced in response to Rho-kinase inhibition. Interestingly, for the compositions that are dominated by actin and α-actinin, the response to Y-27632 was correlated with the actin/α-actinin ratio between these components, such that compositions with higher ratio (A6, B6, C6) were more sensitive and compositions with lower actin-to-α-actinin ratio (A7, B7, C7) were more resistant (Fig. 5A). Upon removal of Y-27632 from the medium, restoration of focal-adhesions and stress-fibers, as well as a reorganization of the lamellipodium, were apparent (Fig. 4 and Supplementary Figs. S1, S2, S3 and S4). Thus, almost all the signatures that were diminished upon Y-27632 treatment were restored (Fig. 5B, signatures in the upper-left quarter are along the diagonal line). Some of the signatures that were resistant to Y-27632 further increased following Y-27632 removal (Fig. 5B, signatures in the upper-right quarter).

## Discussion

In this article we apply multi-component analysis for spatial mapping of compositional signatures along cell adhesion sites and the associated cytoskeleton at a light-microscopy resolution. Compositional diversity is a common feature displayed by many types of sub-cellular structures (e.g. adhesion sites[7], [13], endosomes[14]). Yet, an effective approach for calculating and visualizing these variations has not been developed. Here we demonstrate that 5-color labeling, combined with multi-dimensional clustering of the data, can reveal molecular sub-domains in focal-adhesions and stress-fibers, and highlights the overall molecular reorganization induced in these structures by modulation of Rho-kinase activity.

Simultaneous image analysis of multiple cellular components requires multicolor labeling, quantitative imaging of each individual label, pixel-by-pixel composition analysis, and visualization. Each of these stages can be approached by different methods. Imaging of multiple colors within a single cell can include the use of quantum-dots, which have narrow and tunable emission peaks [15], [16], a broad range of fluorescent dyes, and tagging with a variety of inherently fluorescent proteins in live cells [17]. Microscopes can resolve colors using selective filters, spectral imaging detectors and color un-mixing methods[18]–[20]. A notable alternative for spectral separation is the multi-epitope-ligand cartography, in which the sample is being subjected to repeated cycles of labeling, imaging and bleaching, which enables to image tens of proteins in the same specimen[21]. All these methodologies can, in principle, be combined with the imaging and processing strategy reported here. It should be emphasized that the traditional practice of presenting the entire data in a single image, for example by superposition or ratio imaging [11], [22], is inadequate for imaging of more than 2 colors (Supplementary Fig. S7). Other approaches, based on reducing the dimensionality of the data (e.g. by superimposing all the color channels to a single RGB image [23]) or reducing the intensity resolution (e.g. by binary presentation of above or below an intensity threshold [21]), lead to a major loss of information, and are therefore also incompatible with mapping of quantitative variations in multiple molecular constituents. The labeling approach taken in the present study was based on standard procedures (e.g. combination of primary antibodies from different species, pre-complexing of the primary antibody with tagged secondary Fab fragments and tagging with fluorescent proteins). Careful calibration of the combinations, concentrations and incubation times of the labeling reagents achieved saturated labeling with intensities proportional to local concentrations and a minimal cross-interference between reagents. The fact that the sites of adhesion to an ECM-coated slide are organized along one focal plan facilitates the quantitative imaging of these structures via a single, wide-field, image. The thickness of these structures is below the z-axis resolution of conventional light microscopy, implying similar information for wide-field and confocal microscopy.

The cluster analysis of a very large number of pixels (in the order of one million for a set of 20 images) poses a computational challenge. Hierarchical clustering algorithms require long computation time (*n^2^*(log *n*), where *n* is the number of objects to cluster) and large memory (*n^2^)*. Partitional clustering algorithms (e.g. k-means or fuzzy clustering) are fast but require to pre-determine the total number of clusters and find local, rather than global, clustering optimum. To deal with the difficulty of identifying typical species in large data sets, we applied fast deterministic annealing clustering algorithm, with a computation time of *n*(log *n*)[24]. Following excessive splitting of the pixels population, we used hierarchical clustering, with visual monitoring of spatial coherence, to merge clusters displaying similar compositional signatures.

Cell-matrix adhesions offer an example for the need for, and the power of, multicolor compositional imaging. These sub-cellular structures consist of a large number of components, most of which can interact with multiple partners and, thus, form molecularly heterogeneous networks [7], [8]. Since their molecular heterogeneity is believed to be correlated with their mechanical and signaling functions, it is important to develop a quantitative approach for their compositional mapping. Previous studies revealed variations in the spatial distributions of individual components, based on co-labeling of a low number of components (up to three) (e.g. tensin [10], [11], phospho-paxillin [25], zyxin [12] and integrins [10]). Yet this information is limited, and its functional significance has not yet been elucidated. The molecular mapping described here sheds new light on the molecular diversity of integrin adhesions. It suggests that these structures are regulated at multiple levels, including the whole cell level, the “regional” level, possibly controlled by short-range diffusible factors (e.g. Rho or Rac), and the local level, most likely involving direct signaling from the matrix or the cytoskeleton. This study also shows that individual focal-adhesions often contain internal sub-domains, which are regularly ordered along the axis of the adhesion site. Given the fact that integrin adhesions are dynamic structures, growing in a centripetal direction [26], [27], and that their assembly is induced by mechanical force [9], [10], [12], one may now extend the molecular characterization of integrin adhesions and address the diverse physiological roles of different compositional signatures, and their involvement in the assembly and signaling activities of these adhesion sites.

In conclusion, we demonstrated here that multicolor compositional imaging is a powerful tool for probing the state of molecular organization of sub-cellular structures. It should be emphasized that this approach is rather general, and applicable to any imaging method that can end up with multi-component-per-pixel information, such as mass-spectrometric imaging [28].

## Materials and Methods

### Cells and Y-27632 treatment

Rat embryo fibroblasts (REF52), stably and uniformly expressing β_3_-integrin-GFP, were kindly provided by Dr. C. Ballestrem and Prof. B. Imhof (Centre Medical University, Geneva) and cultured as previously described[26]. β_3_-integrin-GFP had been used in previous studies as a marker for β_3_-integrin localization in fixed and live cells [26], [29]–[32], and was shown to form heterodimers with α_v_-integrin, and to localize at cell-matrix adhesions in an ECM-substrate dependent manner, as the endogenous, wild-type, β_3_-integrin [26]. Cells were maintained in culture in Dulbecco's modified Eagle's medium (DMEM), supplemented with 10% fetal calf serum, glutamine and penicillin. For inhibition of Rho-kinase, cells were plated on glass coverslips, incubated for 24 hours, and then treated with 100 µM Y-27632 (Calbiochem, San Diego, CA, USA) for 3 hours. These conditions resulted in a complete and uniform destruction of focal-adhesions and stress-fibers, which is essential for following their reassembly in different fixed cells shortly after recovery. For recovery, Y-27632-treated cells were rinsed and incubated with drug-free medium for 3, 15 or 60 minutes. At the end of these treatments cells were fixed as described[11].

### Multicolor labeling

For each labeling set, fixed cells were labeled for 4 adhesion components (in addition to the stable expression of β_3_-integrin-GFP) as described in Table 1. The relevant primary antibodies, and the pre-complexed IgG-Fabs, of each set were mixed together and applied to the cells for 1 hour. The cells were then washed with PBS and incubated for 1 hour with a mixture containing the secondary antibodies and CPITC-conjugated phalloidin (Sigma-Aldrich Co.). Finally, the cells were washed with PBS, fixed with 3% paraformaldehyde in PBS for 30 minutes and mounted in Elvanol (Mowiol 4-88, Hoechst, Frankfurt, Germany). The labeling conditions, including the antibody concentrations and incubation times, were calibrated to achieve saturated staining and a minimal cross-interference between the different antibodies.

### Multicolor fluorescent microscopy

Cells were examined using 100x/1.3 objective on IX71 inverted microscope (Olympus Ltd., Tokyo) equipped with Prior Scientific (Cambridge, UK) ProScan filter wheels, shutters and stage controller. Images were recorded using a Quantix back-illuminated CCD camera (Photometrix, Roper Scientific Inc., Tucson AZ). Pixel size is 0.13 µm. A QUINT “zero pixel shift” filters set (Chroma Technology Corp. VT) provided 5 excitation (ex) and emission (em) channels with the following wavelength peaks (±bandwidths): blue (ex: 400±20 nm, em: 450±30 nm), green (ex: 484±30 nm, em: 510±15 nm), red (ex: 548±20 nm, em: 560±14 nm), IR1 (ex: 630±40 nm, em: 670±50 nm) and IR2 (ex: 745±50 nm, em: 770±60 nm). The microscope instrumentation was controlled by software written within the Image Visualization Environment (University of California San Francisco, http://www.msg.ucsf.edu/IVE/). Bleed-through between fluorescent channels were measured using singly labeled samples, and were 0–1% for all pairs, beside 2% and 3.4% from Alexa 750 to the IR1 and red channels respectively. The usage of labeling colors was optimized by having the better reagents (e.g. phalloidin) conjugated with the dimmer fluorophores (e.g. CPITC), and vise versa (e.g. Alexa-555 with Fab).

### Image processing

As was shown before for cell-matrix adhesions[11], [22] and stress-fibers[33] images, it is important to subtract local background from each pixel in order to obtain the correct intensities of these structures. For all images, beside those of actin, local background subtraction was done by high-pass filtration as previously described [11, see Fig. 1 there]. By comparing with other local background estimates, this procedure was found to work well when the integrated intensity of small labeled-structures is much smaller than the integrated intensity of the background, as is the case for cell adhesions. Since this is not the case for actin filaments, these structures were first found by high-pass filtration and intensity threshold, defining actin filaments mask. Then, a high-pass filtration was again applied on the original actin images, but this time excluding from the calculation of the local background pixels falling inside the actin mask[33]. Slightly negative pixels, resulted from the filtration process, were set to zero. A representative set of original images and the corresponding filtered set are included in the Supplementary Materials (Supplementary Fig. S8). The subtraction of local background enables to define for each image a threshold level that segments adhesion structures in the whole image[11]. Thresholds were interactively tuned for each color of each image. The number of pixels corresponding to cell adhesions is too small for robust automated histogram-based thresholding methods, while interactive threshold-setting was found to be robust to small changes and was extensively applied for quantitative studies of these structures (e.g.[11], [22], [33]).

### Data preparation

For each labeling set, five values, corresponding to fluorescence intensities of the five labeled components in the filtered images, are associated with each pixel (“pixel color vectors”: V(Set,Img,Pix(x,y),Col) where Set = A–D labeling sets; Img = 1–20 images; Col = 1–5 labeled components). These vectors were pooled from images of 20 cells for each labeling set (4 cells for each of the 5 treatments), and organized as a matrix with rows corresponding to pixels and five columns corresponding to the labeled components. Rows corresponding to pixels with intensity below threshold levels for all colors (as explained above in “Image processing”) were not included. Each pixel can be considered as a point in a five-dimensional composition space, in which each dimension corresponds to one of the labeled components. Pixels with similar intensity pattern for the five components will be close to each other in such a space, and hence cluster analysis can identify groups of pixels with similar molecular content.

Before clustering the pixels on the basis of their molecular composition, two steps of normalization should be performed. The goal of the first normalization is to give all labeled components equal importance (i.e. “weights”) in the calculation of distances between compositions. Since different components are labeled using different reagents and fluorophores they can have different labeling efficiencies (i.e. emitted light intensity per epitope). Higher labeling efficiency leads to proportionally higher average intensity and differences of intensities between two concentrations of antigens. To make the intensity differences independent of the labeling efficiencies, the intensity of each color (i.e. each column in the matrix) was scaled by the average color intensity: V_s_ = V(Set,Img,Pix(x,y),Col)/ V_avg_(Set, Col) where V_avg_ is the average color intensity for all color values above threshold in all pixels of all images in the labeled set.

The purpose of the second normalization step is to define the composition space by the stoichometric ratio between the components, irrespective of their total amount. Therefore, the length of each pixel vector (i.e. of each row in the matrix) was normalized to one, so that each component will be defined by the fractional intensity (V_sn_ = V_s_(Set,Img,Pix(x,y),Col)/Norm(Set,Img,Pix(x,y)), where Norm(Set,Img,Pix(x,y)) is the square root of the sum of squares for the 5 Color components of each vector V_s_). Without this normalization step the clustering is dominated by total-intensity similarities, and therefore becomes insensitive to stoichometric ratio similarities (data not shown). Associated with each pixel vector, the corresponding image coordinates were saved for a later reconstruction of the compositional images.

### Cluster analysis

Cluster analysis was applied to the rows of the normalized matrix (corresponding to pixels from all 20 multicolor images of one labeling set) in order to find groups of pixels with similar compositions. Pooling pixels from images of all the cells under the different treatments into one data set (of the order of 10^6^ pixels) facilitates quantitative comparison of the results. The clustering task was achieved in two steps. In the first step the data was clustered by a top-down clustering algorithm that can handle large data sets at relatively short computation time. The algorithm splits recursively each population of pixels into two clusters that minimize the sum of Euclidean distances to their centers-of-mass, creating *2^k^* clusters following *k* splits[24]. To ensure sufficient separation between distinct compositions, an excessive splitting was first performed, ending up with 32 clusters. In the second step, these 32 clusters were processed by bottom-up hierarchical clustering. This algorithm finds the two clusters with the shortest Euclidean distance between their centers-of-mass, merges them to a new cluster, and repeats itself recursively till all clusters are merged. The significance of each merging step along this process (i.e. each node in the clustering dendrogram, as in Fig. 2C and Supplementary Fig. S5) was evaluated based on the multi-dimensional distance between the merged clusters (as apparent in the dendrograms in Fig. 2C and Supplementary Fig. S5). The merging was further monitored visually on the resulting compositional images, and approved only if the two clusters involved were also spatially intermixed. This process yielded 8–12 final clusters for each labeling set. Compositional signatures were defined as the average cluster composition (i.e. its center-of-mass). Images presenting the spatial distribution of signatures within cells were created by assigning to each pixel a color indicating its cluster identity. Cluster analyses and construction of composition images were performed by programs written within the Image Visualization Environment (University of California San Francisco, http://www.msg.ucsf.edu/IVE/) and Matlab (The MathWorks, Inc. MA).

## Supporting Information

Supplementary Table S1 Compositional signatures.
(0.14 MB DOC)

Figure S1 Sub-cellular localization of compositional clusters (labeling set A). Sub-cellular localization of the compositional clusters in REF52 cells before (non-treated) or after treatment with the Rho-kinase inhibitor Y-27632 without recovery (Y-27632) or with recovery of different durations. The cells were labeled for vinculin, paxillin, α-actinin, β_3_-integrin and actin (labeling set A) as described in Materials and Methods. Each column shows the 4 cells sampled for the indicated treatment. Each pixel is colored according to its cluster assignment, as indicated by the color-code on the right. The numbers and the colors of the signatures are consistent with Figure 3. Scale bar, 10 µm.
(9.64 MB TIF)

Figure S2 Sub-cellular localization of compositional clusters (labeling set B). As Supplementary Figure S1, with REF52 cells labeled for zyxin, paxillin, α-actinin, β_3_-integrin and actin (labeling set B).
(10.16 MB TIF)

Figure S3 Sub-cellular localization of compositional clusters (labeling set C). As Supplementary Figure S1, with REF52 cells labeled for FAK, paxillin, α-actinin, β_3_-integrin and actin (labeling set C).
(10.06 MB TIF)

Figure S4 Sub-cellular localization of compositional clusters (labeling set D). As Supplementary Figure S1, with REF52 cells labeled for vinculin, paxillin, PY, β_3_-integrin and actin (labeling set D).
(8.49 MB TIF)

Figure S5 Hierarchical merging of over-divided clusters. Bottom-up merging of the 32 over-divided clusters (obtained from the top-down clustering step) for each labeling set, as performed by the hierarchical clustering algorithm. The dendrograms indicate the order of the merging and the distance between the merged clusters. The colored circles mark the nodes that correspond to the final clusters.
(0.46 MB TIF)

Figure S6 Organization of subdomains within single focal-adhesions. Focal-adhesions of non-treated cells, labeled for components of set A (magnified inserts from Supplementary Fig. S1). Note: (i) the diversity between cells, (ii) the high similarity between focal-adhesions in the same cell and (iii) the conserved order between signatures (A2-A3-A9-A8-stress-fibers) along the long focal-adhesions axes. Scale bar, 5 µm.
(1.33 MB TIF)

Figure S7 Visualization of multicolor data by superposition versus compositional imaging. Five-color images of a single cell labeled for vinculin, paxillin, α-actinin, β_3_-integrin, and actin (as shown in Fig. 1) were superimposed with different combinations of artificial colors, as indicated at the bottom (images 1–15). Alternatively (image “CI”), the same 5-color data was subjected to compositional imaging (as shown in Fig. 2). Scale bar, 10 µm.
(8.80 MB TIF)

Figure S8 Example of original and filtered images in 16-bits TIFF format. An example of original images (upper row) and their corresponding filtered images (lower row) of a cell labeled for labeling set A (i.e. from left to right, vinculin, paxillin, α-actinin, β_3_-integrin and actin), in 16-bits TIFF format. This non-compressed presentation pixels retain their true values (as acquired and used for the analysis), and can be viewed using programs that support 16-bits TIFF format, such as ImageJ.
(4.92 MB TIF)
