# Supplementary material for: Quantitative Multicolor Compositional Imaging Resolves Molecular Domains in Cell-Matrix Adhesions
Source: PLoS One. 2008 Apr 2;3(4):e1901. doi: 10.1371/journal.pone.0001901 (PMC2270910; doi:10.1371/journal.pone.0001901)
Supplement: Supplementary Table S1 — Compositional signatures. (0.14 MB DOC) [file pone.0001901.s001.doc]

**Supplementary Table S1.** Compositional signatures.

**Table S1 (A). Cluster average +/- SD for scaled and normalized intensities**

| **set**  **A** | **signature** | **vinculin** | **paxillin** | **α-actinin** | **β3-integrin** | | **actin** |
| --- | --- | --- | --- | --- | --- | --- | --- |
|  | **A1** | 0.07 ± 0.10 | 0.06 ± 0.09 | 0.06 ± 0.07 | 0.03 ± 0.06 | | 0.98 ± 0.03 |
|  | **A2** | 0.23 ± 0.19 | 0.81 ± 0.14 | 0.20 ± 0.21 | 0.22 ± 0.20 | | 0.18 ± 0.17 |
|  | **A3** | 0.27 ± 0.17 | 0.30 ± 0.19 | 0.19 ± 0.19 | 0.78 ± 0.12 | | 0.21 ± 0.16 |
|  | **A4** | 0.13 ± 0.13 | 0.55 ± 0.16 | 0.13 ± 0.15 | 0.08 ± 0.11 | | 0.75 ± 0.14 |
|  | **A5** | 0.06 ± 0.10 | 0.07 ± 0.12 | 0.96 ± 0.05 | 0.05 ± 0.10 | | 0.13 ± 0.11 |
|  | **A6** | 0.07 ± 0.11 | 0.06 ± 0.09 | 0.44 ± 0.12 | 0.03 ± 0.07 | | 0.87 ± 0.07 |
|  | **A7** | 0.10 ± 0.14 | 0.11 ± 0.15 | 0.79 ± 0.09 | 0.08 ± 0.13 | | 0.52 ± 0.12 |
|  | **A8** | 0.41 ± 0.21 | 0.23 ± 0.15 | 0.15 ± 0.15 | 0.32 ± 0.23 | | 0.70 ± 0.13 |
|  | **A9** | 0.55 ± 0.13 | 0.36 ± 0.15 | 0.36 ± 0.20 | 0.44 ± 0.13 | | 0.35 ± 0.16 |
|  | **A10** | 0.93 ± 0.07 | 0.10 ± 0.13 | 0.14 ± 0.15 | 0.10 ± 0.15 | | 0.14 ± 0.14 |
| **set**  **B** | **signature** | **zyxin** | **paxillin** | **α-actinin** | **β3-integrin** | **actin** | |
|  | **B1** | 0.06 ± 0.09 | 0.05 ± 0.08 | 0.07 ± 0.10 | 0.02 ± 0.05 | 0.98 ± 0.02 | |
|  | **B2** | 0.22 ± 0.16 | 0.75 ± 0.17 | 0.19 ± 0.19 | 0.36 ± 0.28 | 0.16 ± 0.16 | |
|  | **B3** | 0.12 ± 0.10 | 0.25 ± 0.15 | 0.15 ± 0.18 | 0.88 ± 0.09 | 0.18 ± 0.17 | |
|  | **B4** | 0.12 ± 0.13 | 0.57 ± 0.15 | 0.09 ± 0.12 | 0.05 ± 0.07 | 0.76 ± 0.13 | |
|  | **B5** | 0.10 ± 0.15 | 0.08 ± 0.14 | 0.95 ± 0.06 | 0.04 ± 0.10 | 0.12 ± 0.11 | |
|  | **B6** | 0.14 ± 0.15 | 0.08 ± 0.10 | 0.49 ± 0.13 | 0.03 ± 0.08 | 0.82 ± 0.07 | |
|  | **B7** | 0.12 ± 0.12 | 0.14 ± 0.16 | 0.80 ± 0.09 | 0.09 ± 0.15 | 0.48 ± 0.12 | |
|  | **B8** | 0.20 ± 0.16 | 0.25 ± 0.16 | 0.11 ± 0.11 | 0.48 ± 0.16 | 0.74 ± 0.12 | |
|  | **B9** | 0.69 ± 0.17 | 0.35 ± 0.20 | 0.23 ± 0.19 | 0.30 ± 0.22 | 0.26 ± 0.18 | |
|  | **B10** | 0.55 ± 0.15 | 0.20 ± 0.15 | 0.14 ± 0.12 | 0.07 ± 0.09 | 0.74 ± 0.12 | |
|  | **B11** | 0.53 ± 0.14 | 0.06 ± 0.08 | 0.69 ± 0.11 | 0.03 ± 0.06 | 0.42 ± 0.16 | |
| **set**  **C** | **signature** | **FAK** | **paxillin** | **α-actinin** | **β3-integrin** | **actin** | |
|  | **C1** | 0.06 ± 0.10 | 0.06 ± 0.10 | 0.06 ± 0.08 | 0.03 ± 0.07 | 0.98 ± 0.03 | |
|  | **C2** | 0.23 ± 0.21 | 0.87 ± 0.11 | 0.08 ± 0.14 | 0.20 ± 0.21 | 0.11 ± 0.13 | |
|  | **C3** | 0.18 ± 0.16 | 0.37 ± 0.18 | 0.07 ± 0.10 | 0.85 ± 0.09 | 0.14 ± 0.13 | |
|  | **C4** | 0.17 ± 0.15 | 0.60 ± 0.16 | 0.12 ± 0.15 | 0.11 ± 0.12 | 0.70 ± 0.14 | |
|  | **C5** | 0.03 ± 0.05 | 0.04 ± 0.07 | 0.98 ± 0.02 | 0.02 ± 0.05 | 0.12 ± 0.11 | |
|  | **C6** | 0.07 ± 0.10 | 0.07 ± 0.11 | 0.44 ± 0.12 | 0.03 ± 0.07 | 0.87 ± 0.07 | |
|  | **C7** | 0.04 ± 0.07 | 0.07 ± 0.10 | 0.81 ± 0.08 | 0.03 ± 0.06 | 0.55 ± 0.11 | |
|  | **C8** | 0.25 ± 0.17 | 0.45 ± 0.19 | 0.30 ± 0.22 | 0.52 ± 0.18 | 0.41 ± 0.23 | |
|  | **C9** | 0.15 ± 0.10 | 0.31 ± 0.15 | 0.80 ± 0.10 | 0.20 ± 0.18 | 0.30 ± 0.19 | |
|  | **C10** | 0.44 ± 0.16 | 0.07 ± 0.10 | 0.79 ± 0.13 | 0.03 ± 0.06 | 0.28 ± 0.22 | |
|  | **C11** | 0.63 ± 0.17 | 0.13 ± 0.15 | 0.11 ± 0.15 | 0.05 ± 0.10 | 0.68 ± 0.16 | |
|  | **C12** | 0.91 ± 0.10 | 0.12 ± 0.15 | 0.14 ± 0.18 | 0.08 ± 0.17 | 0.12 ± 0.14 | |
| **set**  **D** | **signature** | **vinculin** | **paxillin** | **PY** | **β3-integrin** | **actin** | |
|  | **D1** | 0.10 ± 0.15 | 0.08 ± 0.11 | 0.07 ± 0.12 | 0.04 ± 0.09 | 0.96 ± 0.06 | |
|  | **D2** | 0.20 ± 0.19 | 0.85 ± 0.11 | 0.16 ± 0.17 | 0.23 ± 0.19 | 0.16 ± 0.15 | |
|  | **D3** | 0.22 ± 0.15 | 0.42 ± 0.19 | 0.18 ± 0.16 | 0.78 ± 0.12 | 0.15 ± 0.12 | |
|  | **D4** | 0.10 ± 0.11 | 0.58 ± 0.13 | 0.08 ± 0.11 | 0.07 ± 0.12 | 0.75 ± 0.13 | |
|  | **D5** | 0.30 ± 0.17 | 0.33 ± 0.14 | 0.20 ± 0.14 | 0.60 ± 0.14 | 0.55 ± 0.14 | |
|  | **D6** | 0.76 ± 0.15 | 0.25 ± 0.20 | 0.16 ± 0.15 | 0.34 ± 0.24 | 0.20 ± 0.17 | |
|  | **D7** | 0.55 ± 0.15 | 0.51 ± 0.14 | 0.16 ± 0.14 | 0.13 ± 0.12 | 0.54 ± 0.16 | |
|  | **D8** | 0.18 ± 0.21 | 0.17 ± 0.20 | 0.71 ± 0.18 | 0.10 ± 0.15 | 0.45 ± 0.27 | |

**Table S1 (B). Cluster average +/- SD for scaled intensities (shown in Fig. 3)**

| **set**  **A** | **signature** | **vinculin** | **paxillin** | **α-actinin** | **β3-integrin** | | **actin** |
| --- | --- | --- | --- | --- | --- | --- | --- |
|  | **A1** | 0.08 ± 0.12 | 0.07 ± 0.11 | 0.08 ± 0.10 | 0.04 ± 0.08 | | 1.19 ± 0.03 |
|  | **A2** | 0.33 ± 0.27 | 0.93 ± 0.16 | 0.25 ± 0.27 | 0.32 ± 0.29 | | 0.24 ± 0.23 |
|  | **A3** | 0.55 ± 0.35 | 0.57 ± 0.36 | 0.41 ± 0.41 | 1.42 ± 0.22 | | 0.45 ± 0.34 |
|  | **A4** | 0.14 ± 0.13 | 0.47 ± 0.14 | 0.13 ± 0.15 | 0.08 ± 0.12 | | 0.66 ± 0.12 |
|  | **A5** | 0.07 ± 0.14 | 0.07 ± 0.13 | 1.29 ± 0.07 | 0.09 ± 0.18 | | 0.20 ± 0.17 |
|  | **A6** | 0.09 ± 0.13 | 0.08 ± 0.12 | 0.54 ± 0.15 | 0.05 ± 0.10 | | 1.07 ± 0.08 |
|  | **A7** | 0.14 ± 0.21 | 0.14 ± 0.19 | 1.15 ± 0.14 | 0.17 ± 0.29 | | 0.75 ± 0.18 |
|  | **A8** | 0.72 ± 0.36 | 0.45 ± 0.29 | 0.32 ± 0.31 | 0.67 ± 0.48 | | 1.28 ± 0.24 |
|  | **A9** | 1.35 ± 0.32 | 0.89 ± 0.36 | 0.87 ± 0.48 | 1.12 ± 0.33 | | 0.89 ± 0.41 |
|  | **A10** | 1.28 ± 0.10 | 0.13 ± 0.17 | 0.20 ± 0.22 | 0.12 ± 0.19 | | 0.20 ± 0.20 |
| **set**  **B** | **signature** | **zyxin** | **paxillin** | **α-actinin** | **β3-integrin** | **actin** | |
|  | **B1** | 0.08 ± 0.13 | 0.06 ± 0.10 | 0.09 ± 0.14 | 0.02 ± 0.06 | 1.22 ± 0.03 | |
|  | **B2** | 0.30 ± 0.22 | 0.78 ± 0.18 | 0.21 ± 0.21 | 0.49 ± 0.37 | 0.17 ± 0.17 | |
|  | **B3** | 0.20 ± 0.18 | 0.38 ± 0.22 | 0.29 ± 0.35 | 1.20 ± 0.12 | 0.36 ± 0.34 | |
|  | **B4** | 0.11 ± 0.12 | 0.39 ± 0.10 | 0.07 ± 0.09 | 0.04 ± 0.07 | 0.54 ± 0.09 | |
|  | **B5** | 0.13 ± 0.19 | 0.11 ± 0.19 | 1.23 ± 0.08 | 0.09 ± 0.21 | 0.19 ± 0.18 | |
|  | **B6** | 0.20 ± 0.22 | 0.10 ± 0.13 | 0.60 ± 0.16 | 0.05 ± 0.12 | 1.05 ± 0.09 | |
|  | **B7** | 0.23 ± 0.24 | 0.23 ± 0.26 | 1.37 ± 0.15 | 0.27 ± 0.44 | 0.79 ± 0.20 | |
|  | **B8** | 0.38 ± 0.31 | 0.36 ± 0.22 | 0.23 ± 0.24 | 0.76 ± 0.26 | 1.18 ± 0.20 | |
|  | **B9** | 1.79 ± 0.44 | 0.85 ± 0.49 | 0.57 ± 0.46 | 0.90 ± 0.66 | 0.72 ± 0.50 | |
|  | **B10** | 0.87 ± 0.24 | 0.37 ± 0.28 | 0.26 ± 0.22 | 0.17 ± 0.23 | 1.20 ± 0.20 | |
|  | **B11** | 0.57 ± 0.15 | 0.09 ± 0.11 | 0.74 ± 0.12 | 0.04 ± 0.10 | 0.48 ± 0.18 | |
| **set**  **C** | **signature** | **FAK** | **paxillin** | **α-actinin** | **β3-integrin** | **actin** | |
|  | **C1** | 0.10 ± 0.17 | 0.08 ± 0.14 | 0.11 ± 0.14 | 0.05 ± 0.11 | 1.31 ± 0.04 | |
|  | **C2** | 0.26 ± 0.24 | 0.85 ± 0.10 | 0.08 ± 0.14 | 0.25 ± 0.26 | 0.13 ± 0.15 | |
|  | **C3** | 0.28 ± 0.25 | 0.54 ± 0.27 | 0.12 ± 0.16 | 1.08 ± 0.12 | 0.24 ± 0.22 | |
|  | **C4** | 0.21 ± 0.18 | 0.59 ± 0.15 | 0.16 ± 0.20 | 0.16 ± 0.17 | 0.73 ± 0.15 | |
|  | **C5** | 0.05 ± 0.08 | 0.05 ± 0.09 | 1.24 ± 0.03 | 0.03 ± 0.08 | 0.18 ± 0.16 | |
|  | **C6** | 0.11 ± 0.16 | 0.11 ± 0.15 | 0.56 ± 0.16 | 0.04 ± 0.10 | 1.15 ± 0.09 | |
|  | **C7** | 0.07 ± 0.10 | 0.09 ± 0.13 | 0.99 ± 0.10 | 0.04 ± 0.09 | 0.66 ± 0.14 | |
|  | **C8** | 0.56 ± 0.36 | 0.90 ± 0.38 | 0.66 ± 0.48 | 1.12 ± 0.39 | 0.92 ± 0.50 | |
|  | **C9** | 0.31 ± 0.22 | 0.51 ± 0.25 | 1.54 ± 0.19 | 0.48 ± 0.41 | 0.60 ± 0.38 | |
|  | **C10** | 0.45 ± 0.16 | 0.08 ± 0.11 | 0.84 ± 0.14 | 0.04 ± 0.08 | 0.31 ± 0.24 | |
|  | **C11** | 0.61 ± 0.16 | 0.13 ± 0.15 | 0.13 ± 0.18 | 0.06 ± 0.11 | 0.67 ± 0.16 | |
|  | **C12** | 1.00 ± 0.11 | 0.12 ± 0.15 | 0.16 ± 0.21 | 0.10 ± 0.20 | 0.17 ± 0.19 | |
| **set**  **D** | **signature** | **vinculin** | **paxillin** | **PY** | **β3-integrin** | **actin** | |
|  | **D1** | 0.12 ± 0.19 | 0.10 ± 0.14 | 0.10 ± 0.16 | 0.05 ± 0.11 | 1.13 ± 0.07 | |
|  | **D2** | 0.35 ± 0.34 | 1.07 ± 0.14 | 0.28 ± 0.29 | 0.38 ± 0.32 | 0.21 ± 0.20 | |
|  | **D3** | 0.45 ± 0.31 | 0.79 ± 0.36 | 0.36 ± 0.33 | 1.33 ± 0.21 | 0.31 ± 0.24 | |
|  | **D4** | 0.10 ± 0.12 | 0.46 ± 0.10 | 0.09 ± 0.13 | 0.09 ± 0.15 | 0.58 ± 0.10 | |
|  | **D5** | 0.71 ± 0.39 | 0.74 ± 0.30 | 0.47 ± 0.34 | 1.31 ± 0.30 | 1.19 ± 0.31 | |
|  | **D6** | 1.48 ± 0.28 | 0.68 ± 0.55 | 0.47 ± 0.46 | 0.92 ± 0.65 | 0.40 ± 0.34 | |
|  | **D7** | 0.82 ± 0.23 | 0.78 ± 0.21 | 0.32 ± 0.28 | 0.26 ± 0.24 | 0.80 ± 0.25 | |
|  | **D8** | 0.26 ± 0.31 | 0.23 ± 0.26 | 0.81 ± 0.21 | 0.14 ± 0.23 | 0.49 ± 0.30 | |

The scaled and normalized intensities ($V_{\text{sn}}$, Table S1, A) and the scaled intensities ($V_{s}$, Table S1, B) were averaged over all pixels of the same cluster (i.e. signature) and are given with standard deviations. In Table S1, B the standard deviations are the normalized intensities standard deviations multiplied by the ratio between the corresponding ratio of non-normalized and normalized average intensities, , of each cluster. As explained in the text, these standard deviations indicate composition variations rather than intensity variations.
