# Supplementary figures and images for: Quantitative Multicolor Compositional Imaging Resolves Molecular Domains in Cell-Matrix Adhesions
Source: PLoS One. 2008 Apr 2;3(4):e1901. doi: 10.1371/journal.pone.0001901 (PMC2270910; doi:10.1371/journal.pone.0001901)

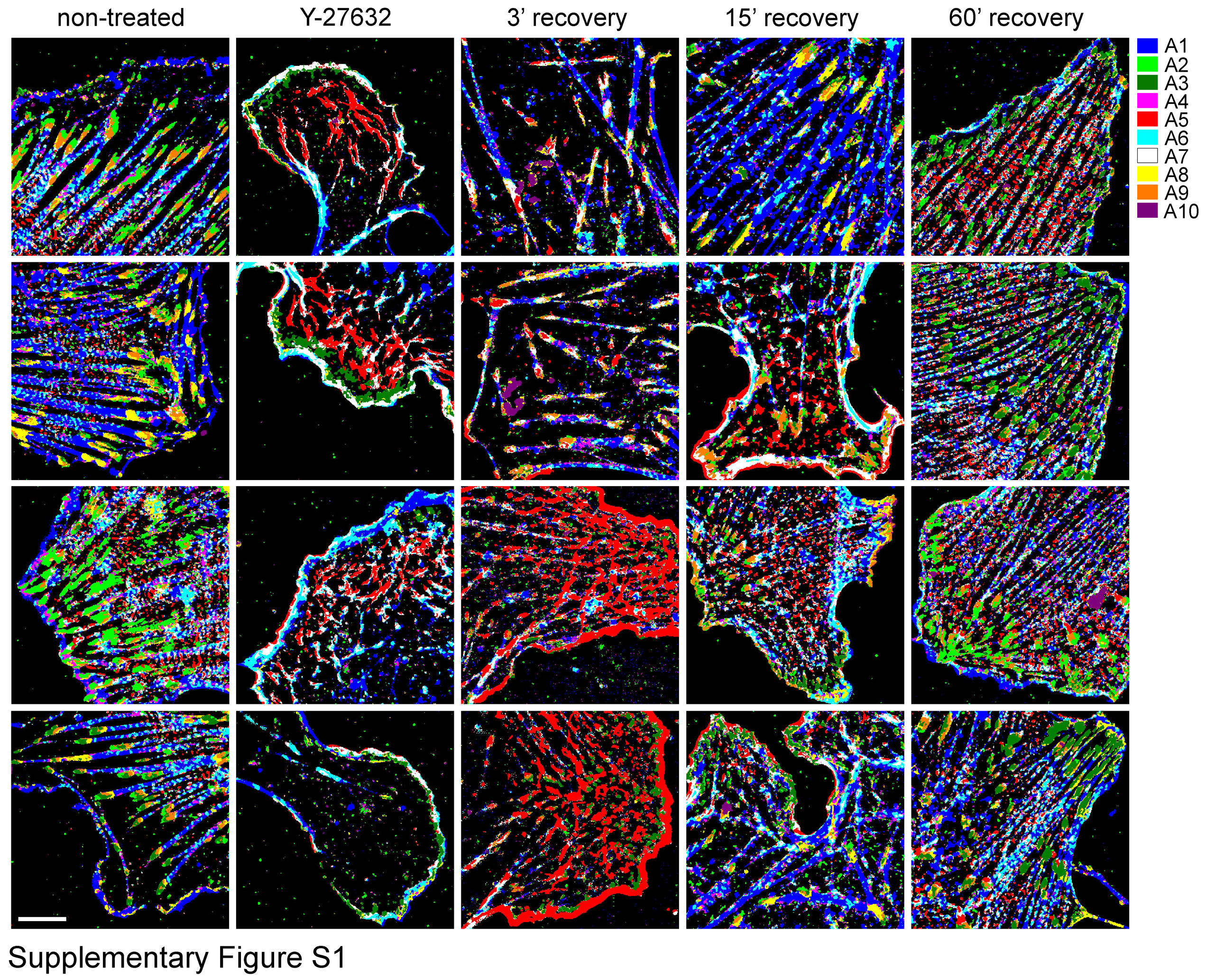

Supplement: Figure S1 — Sub-cellular localization of compositional clusters (labeling set A). Sub-cellular localization of the compositional clusters in REF52 cells before (non-treated) or after treatment with the Rho-kinase inhibitor Y-27632 without recovery (Y-27632) or with recovery of different durations. The cells were labeled for vinculin, paxillin, α-actinin, β3-integrin and actin (labeling set A) as described in Materials and Methods. Each column shows the 4 cells sampled for the indicated treatment. Each pixel is colored according to its cluster assignment, as indicated by the color-code on the right. The numbers and the colors of the signatures are consistent with Figure 3. Scale bar, 10 µm. (9.64 MB TIF) [file pone.0001901.s002.tif]

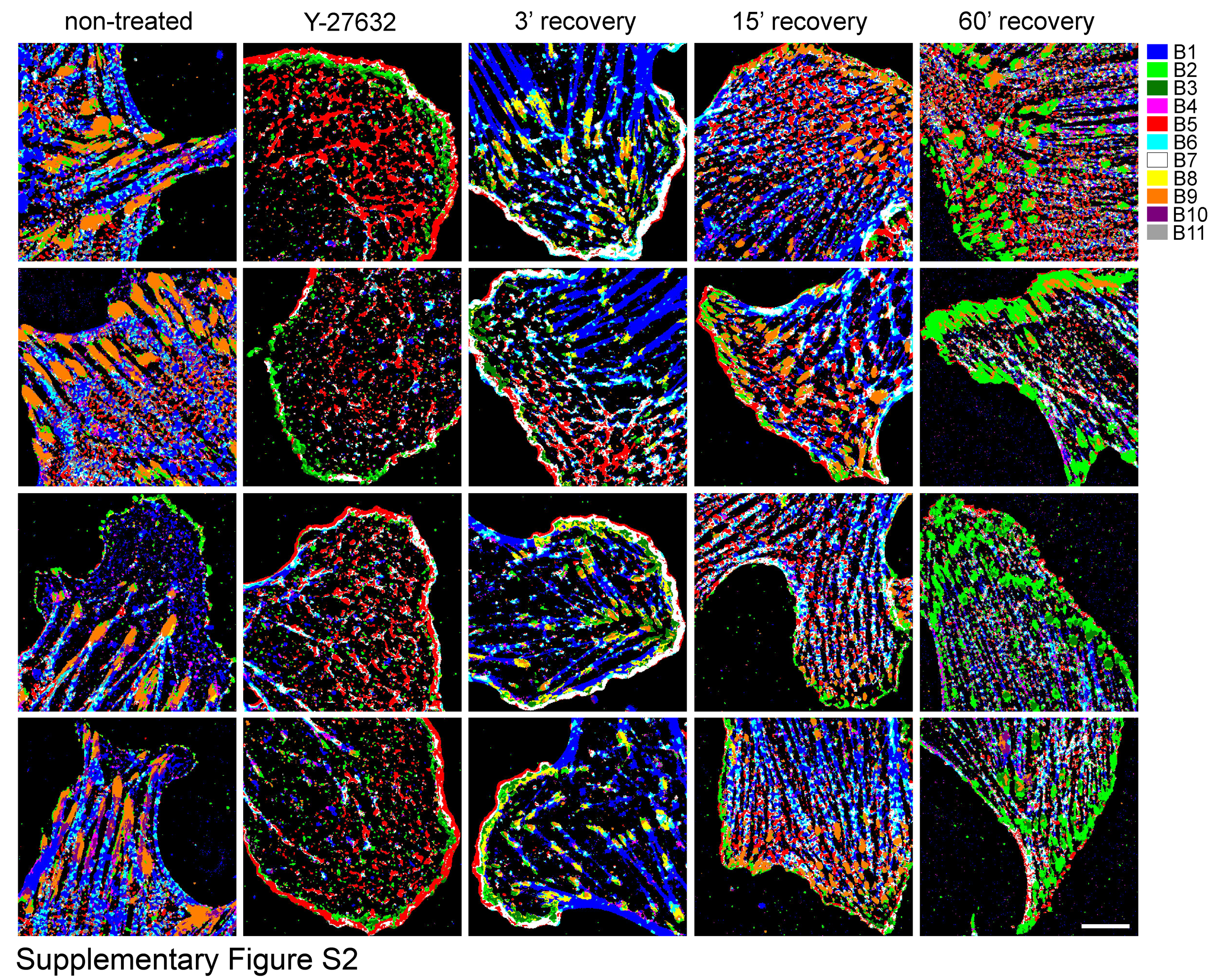

Supplement: Figure S2 — Sub-cellular localization of compositional clusters (labeling set B). As Supplementary Figure S1, with REF52 cells labeled for zyxin, paxillin, α-actinin, β3-integrin and actin (labeling set B). (10.16 MB TIF) [file pone.0001901.s003.tif]

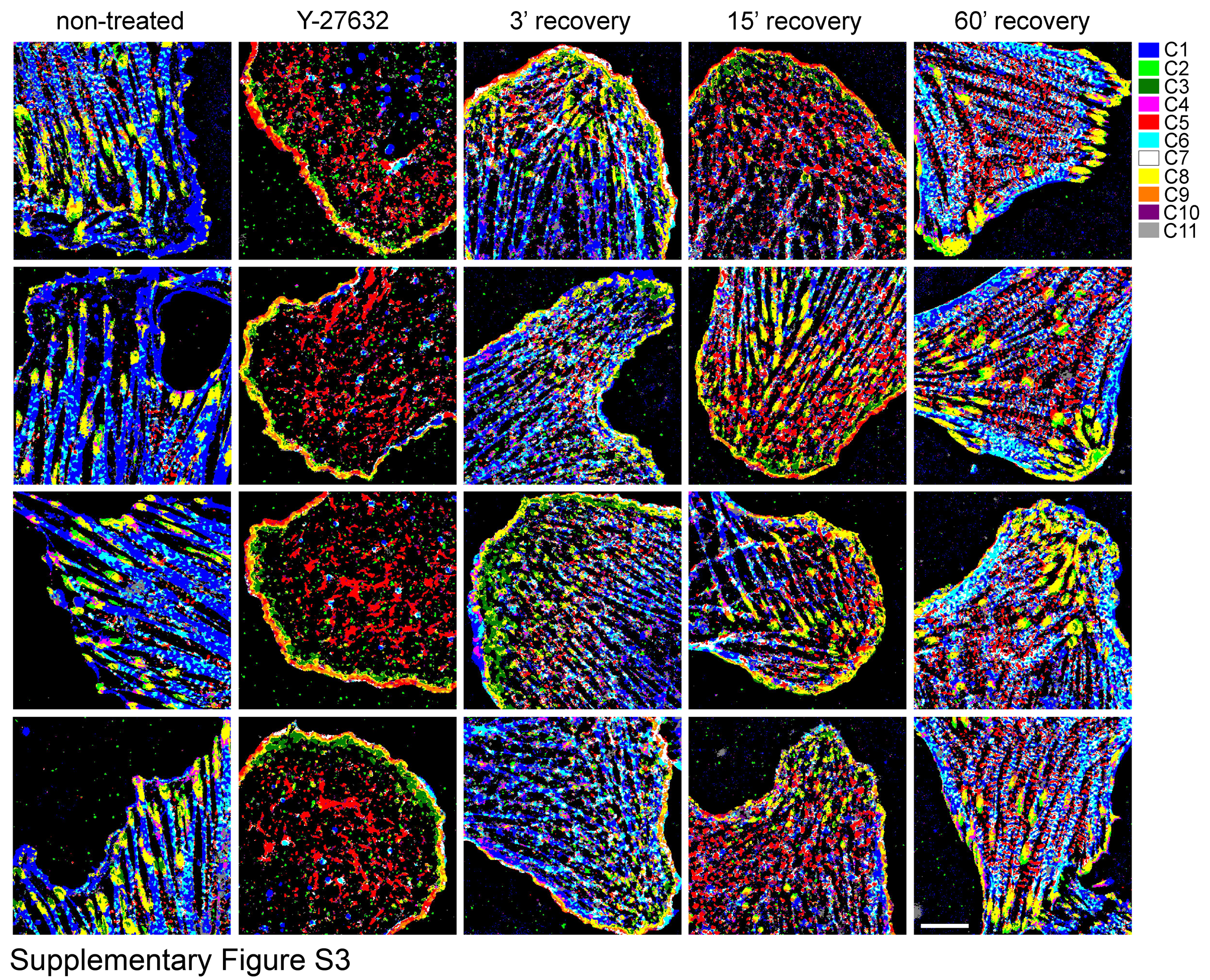

Supplement: Figure S3 — Sub-cellular localization of compositional clusters (labeling set C). As Supplementary Figure S1, with REF52 cells labeled for FAK, paxillin, α-actinin, β3-integrin and actin (labeling set C). (10.06 MB TIF) [file pone.0001901.s004.tif]

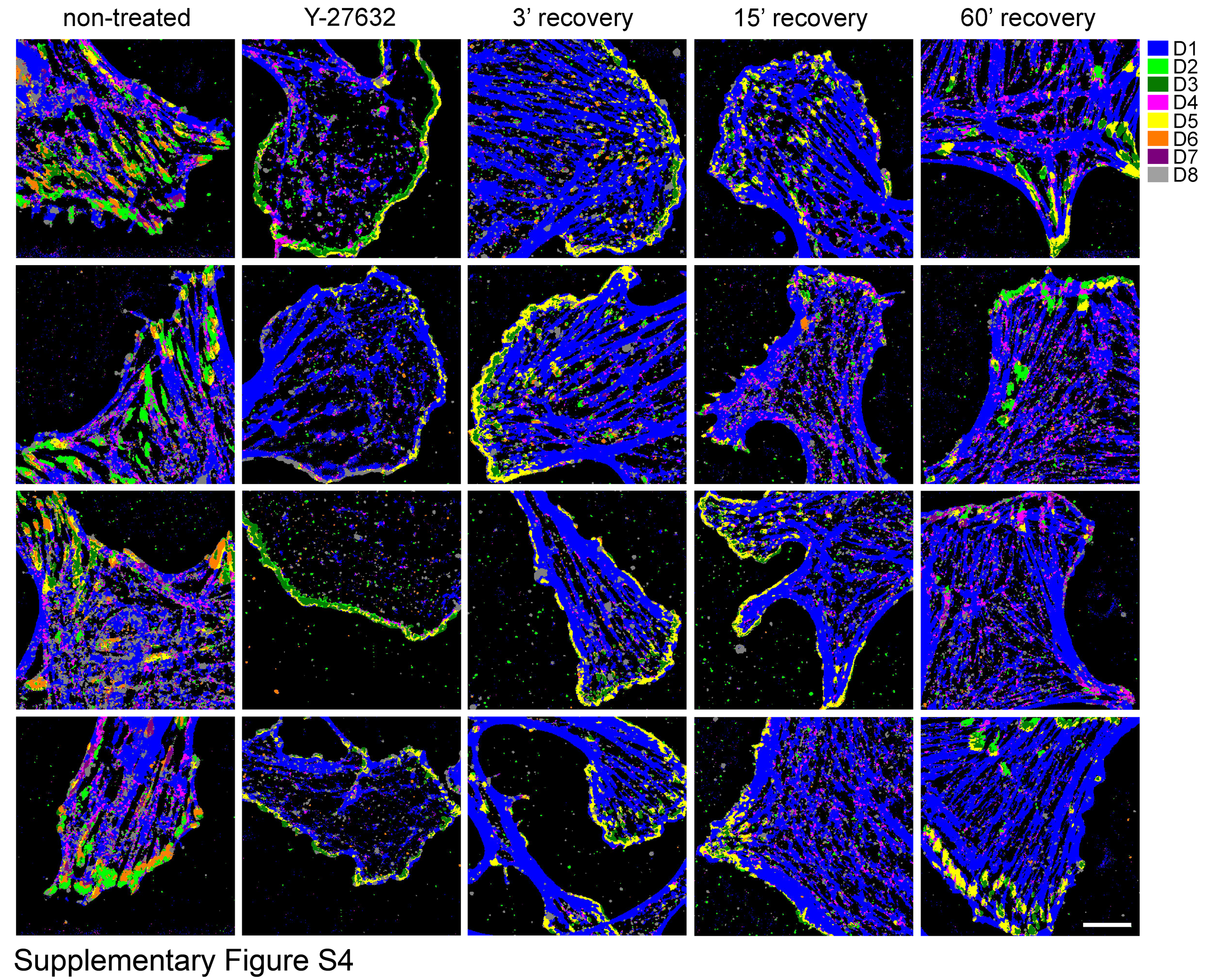

Supplement: Figure S4 — Sub-cellular localization of compositional clusters (labeling set D). As Supplementary Figure S1, with REF52 cells labeled for vinculin, paxillin, PY, β3-integrin and actin (labeling set D). (8.49 MB TIF) [file pone.0001901.s005.tif]

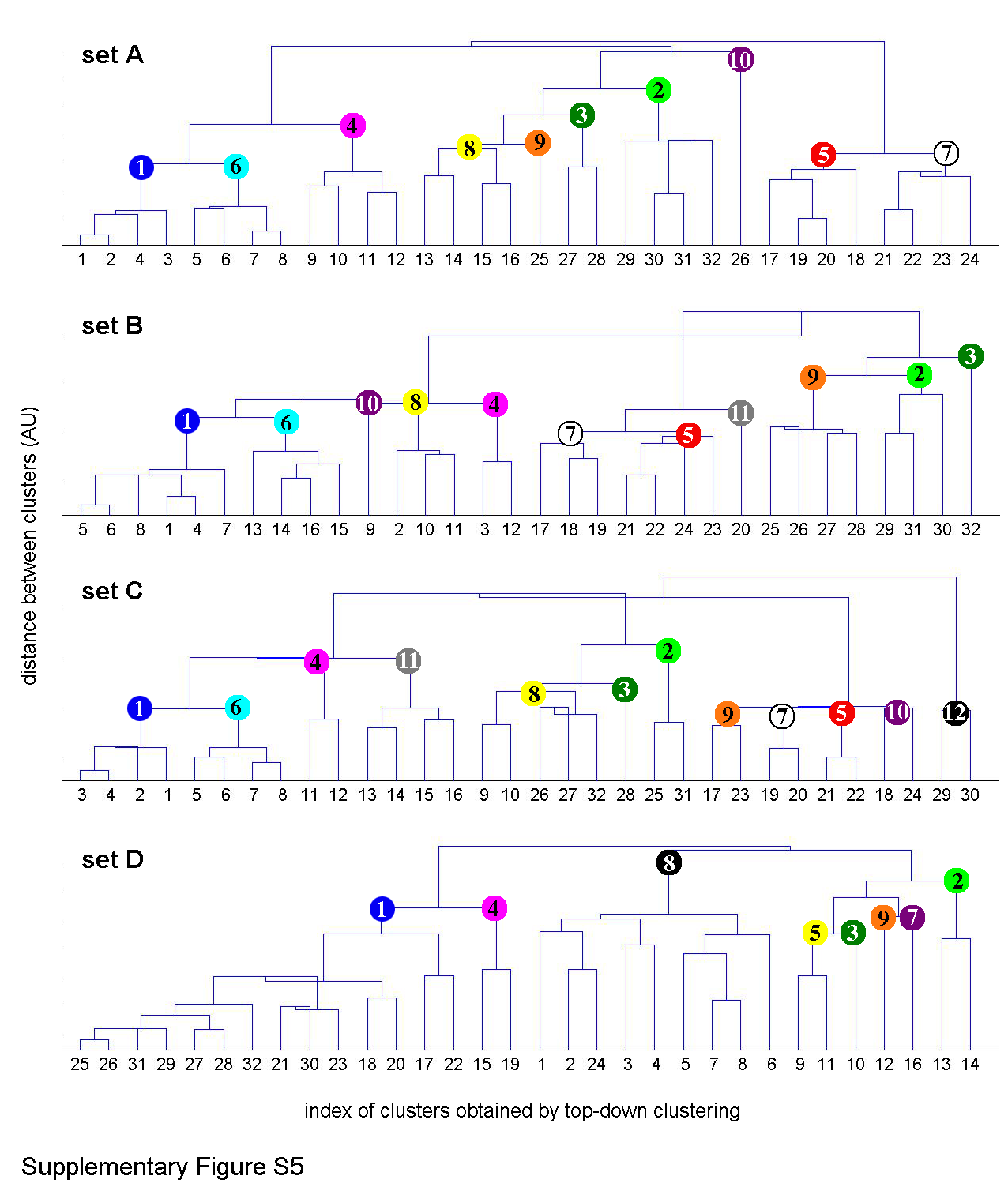

Supplement: Figure S5 — Hierarchical merging of over-divided clusters. Bottom-up merging of the 32 over-divided clusters (obtained from the top-down clustering step) for each labeling set, as performed by the hierarchical clustering algorithm. The dendrograms indicate the order of the merging and the distance between the merged clusters. The colored circles mark the nodes that correspond to the final clusters. (0.46 MB TIF) [file pone.0001901.s006.tif]

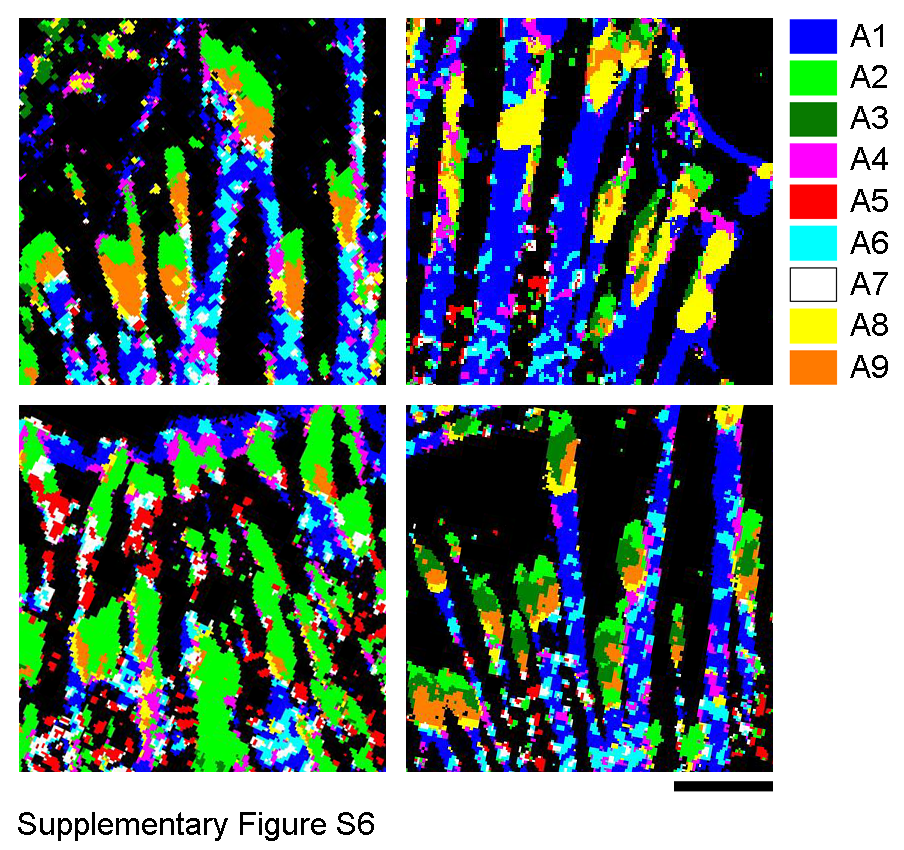

Supplement: Figure S6 — Organization of subdomains within single focal-adhesions. Focal-adhesions of non-treated cells, labeled for components of set A (magnified inserts from Supplementary Fig. S1). Note: (i) the diversity between cells, (ii) the high similarity between focal-adhesions in the same cell and (iii) the conserved order between signatures (A2-A3-A9-A8-stress-fibers) along the long focal-adhesions axes. Scale bar, 5 µm. (1.33 MB TIF) [file pone.0001901.s007.tif]

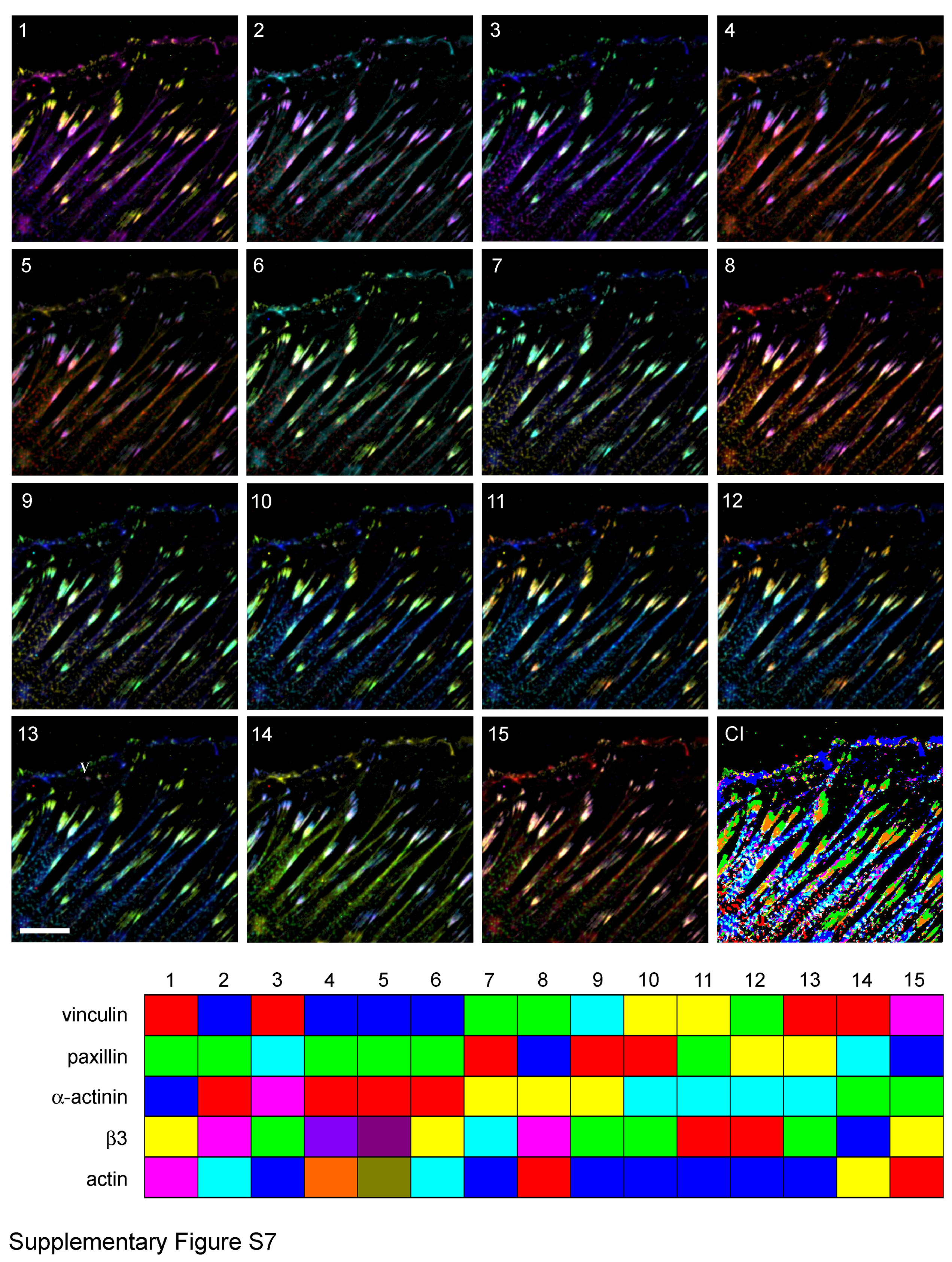

Supplement: Figure S7 — Visualization of multicolor data by superposition versus compositional imaging. Five-color images of a single cell labeled for vinculin, paxillin, α-actinin, β3-integrin, and actin (as shown in Fig. 1) were superimposed with different combinations of artificial colors, as indicated at the bottom (images 1–15). Alternatively (image “CI”), the same 5-color data was subjected to compositional imaging (as shown in Fig. 2). Scale bar, 10 µm. (8.80 MB TIF) [file pone.0001901.s008.tif]
